# Dietary geranylgeraniol can limit the activity of pitavastatin as a potential treatment for drug-resistant ovarian cancer

**DOI:** 10.1038/s41598-017-05595-4

**Published:** 2017-07-14

**Authors:** Elizabeth de Wolf, Marwan Ibrahim Abdullah, Stefanie M. Jones, Karen Menezes, Darren M. Moss, Falko P. Drijfhout, Sarah R. Hart, Clare Hoskins, Euan A. Stronach, Alan Richardson

**Affiliations:** 1 Institute for Science and Technology in Medicine, School of Pharmacy, Keele University, Newcastle-under-Lyme, UK; 2 Faculty of Medicine, Department of Surgery & Cancer, Imperial College, London, UK; 3 Chemistry Department, Keele University, Newcastle-under-Lyme, UK; 4 Institute for Science and Technology in Medicine, School of Medicine, Keele University, Newcastle-under-Lyme, UK

## Abstract

Pre-clinical and retrospective studies of patients using statins to reduce plasma cholesterol have suggested that statins may be useful to treat cancer. However, prospective clinical trials have yet to demonstrate significant efficacy. We have previously shown that this is in part because a hydrophobic statin with a long half-life is necessary. Pitavastatin, the only statin with this profile, has not undergone clinical evaluation in oncology. The target of pitavastatin, hydroxymethylglutarate coenzyme-A reductase (HMGCR), was found to be over-expressed in all ovarian cancer cell lines examined and upregulated by mutated *TP53*, a gene commonly altered in ovarian cancer. Pitavastatin-induced apoptosis was blocked by geranylgeraniol and mevalonate, products of the HMGCR pathway, confirming that pitavastatin causes cell death through inhibition of HMGCR. Solvent extracts of human and mouse food were also able to block pitavastatin-induced apoptosis, suggesting diet might influence the outcome of clinical trials. When nude mice were maintained on a diet lacking geranylgeraniol, oral pitavastatin caused regression of Ovcar-4 tumour xenografts. However, when the animal diet was supplemented with geranylgeraniol, pitavastatin failed to prevent tumour growth. This suggests that a diet containing geranylgeraniol can limit the anti-tumour activity of pitavastatin and diet should be controlled in clinical trials of statins.

## Introduction

Ovarian cancer remains an inadequately treated disease. Most ovarian cancer patients initially respond to chemotherapy but unfortunately the majority of patients relapse with a recurrence of the disease which ultimately becomes resistant to chemotherapy. Although molecularly-targeted therapies are beginning to make an impact, for example, PARP inhibitors^1^, currently only 40% of patients survive beyond 5 years^2^. New therapies are needed and one solution to this is to redeploy drugs which are currently used to treat other diseases.

Statins reduce plasma cholesterol by inhibiting hydroxymethylglutarate Coenzyme-A reductase (HMGCR) to prevent the synthesis of mevalonate, a precursor to cholesterol, and the isoprenoids farnesol and geranylgeraniol^3^. These isoprenoids are used to covalently modify several small GTPases and anchor them to the cell membrane. Membrane localization is necessary for the function of many small GTPases and because several small GTPases are oncogenes, there is a mechanistic rationale for interfering with their prenylation in cancer. *HMGCR* is itself considered a metabolic oncogene. It promotes tumour growth and it co-operates with Ras to transform cells in colony forming assays^4^. Recent data suggest that expression of *HMGCR* can be increased in cancer cells by wild-type^5^ or gain-of-function mutants of *TP53* ^6^. Mutation of *TP53* can often lead to increased p53 protein because of the loss of the MDM-2 dependant negative feedback loop that normally controls p53 protein levels. Considering that mutation of *TP53* is an almost invariant feature of ovarian cancer^7^, *HMGCR* expression can be expected to be deregulated in a significant proportion of ovarian cancers. This hypothesis is supported by immunohistochemical studies which have identified HMGCR expression in 65% of ovarian cancers^8^ as well as in other cancer types^9–11^. In addition, inhibition of HMGCR by statins alters the expression of several genes contributing to carcinogenesis^12^. Taken together, these data suggest that inhibition of HMGCR by statins may be beneficial in the treatment of cancer. In support of this, epidemiological studies have found a link between statin use to control hypercholesterolemia and reduced mortality from various cancers^13^, including ovarian cancer^14^.

Despite these encouraging observations, prospective clinical trials repurposing statins to treat cancer have so far mostly been unimpressive (reviewed in ref. 13). The lack of success of statins in prospective clinical trials in various cancers is likely to have multiple causes. Many of these trials have used the dose of a statin which is typically used to treat hypercholesterolemia^15–19^. Such doses result in a plasma concentration of drug that falls well below the concentration we have shown to be necessary to cause cell death *in vitro* ^20^. However, clinical trials using doses close to the maximum tolerated dose have also not succeeded^21–25^. Some clinical trials have also used the once daily dosing interval commonly used to treat hypercholesterolaemia^15–17, 19, 26–28^. In contrast, we have shown that continual inhibition of HMGCR is necessary to kill cells *in vitro* because 12 hour cycles of simvastatin exposure interspersed with 12 hours “drug holiday” completely abrogates the cytotoxic activity of simvastatin *in vitro* ^20^. This suggests that short half-life statins (e.g. simvastatin, t_½_ = 2–3 hours)^29^ given once daily to patients are unlikely to maintain adequate inhibition of HMGCR to be effective, particularly if high doses are not used. Hydrophobic statins may also be preferred because they are more potent inducers of cell death than the hydrophilic statins^20^. Pitavastatin is unique because it is the only statin which combines a suitably long metabolic half-life^29^ (11 hours) to allow continual inhibition of HMGCR by twice daily dosing, with a lipophilic structure which renders it a potent inhibitor of HMGCR. Clinical trials of pitavastatin in cancer have yet to be reported, yet the arguments presented above suggest it is the statin most likely to efficacious in the treatment of cancer.

To justify clinical trials of pitavastatin in ovarian cancer, we have evaluated its activity against a panel of ovarian cancer cell lines. We show that pitavastatin induces apoptosis in ovarian cancer cells. However, this can be suppressed by exposure of the cells to geranylgeraniol as well as by extracts from mouse chow and human foodstuffs, raising the possibility that dietary isoprenoids may impede the effectiveness of statins in animal studies and in clinical studies in oncology. Ovcar-4 ovarian cancer xenografts in mice fed a diet lacking geranylgeraniol regressed when treated with pitavastatin. However, when mice received a diet supplemented with geranylgeraniol, the tumour continued to grow. This suggests that patients’ diet must be controlled for statins to be maximally effective in the treatment of cancer. More generally, clinical trials of drugs which affect metabolic processes may need to take into account patients’ diet.

## Results

**Figure 1:**
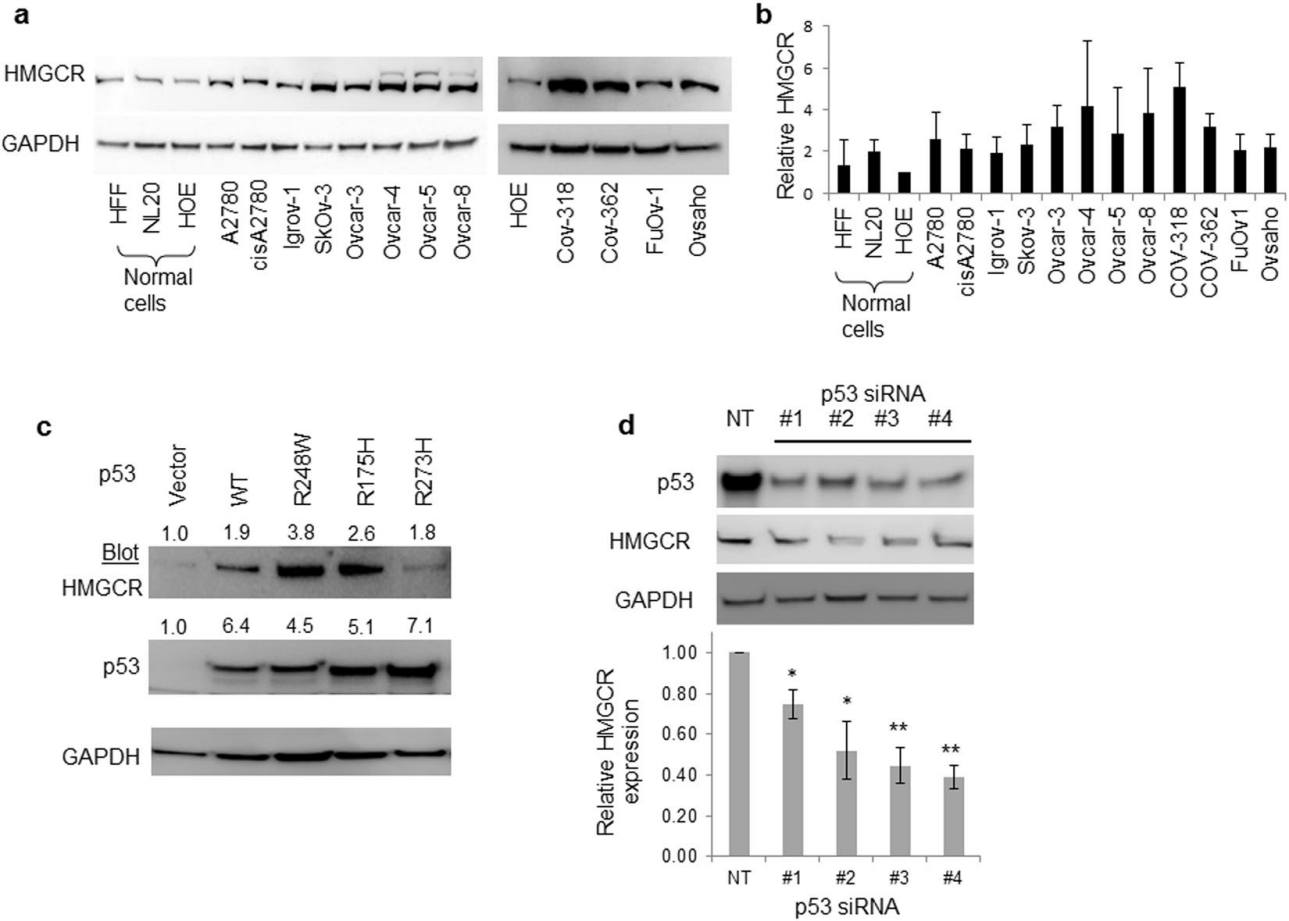
HMGCR is over-expressed in ovarian cancer cells and regulated by *TP53*. (**a**) The expression of HMGCR was measured by immunoblotting and quantified (**b**) in a panel of ovarian cancer cells and compared to non-cancerous cells including normal human ovarian epithelia (HOE), human foreskin fibroblasts (HFF) normal lung epithelia (NL20). (**c**) Wild (WT) and *TP53* variants were over-expressed in SkOv-3 cells and p53 and HMGCR measured by immunoblotting. The numbers above the blots show the mean change in protein normalized to GAPDH (n = 3 lysates) and expressed as ratio of that measured in cells transfected with the vector. D. Ovcar-3 cells were transfected with non-targeting (NT) siRNA or 4 different p53 siRNA (#1, #2, #3, #4) and reduction in p53 and HMGCR protein confirmed by immunoblotting (n = 3 independent transfections). Expression was quantified and normalized to that in normal cells (mean ± S.D, n = 3, *paired *t*-test, *P* < 0.05, compared to cells transfected with vector). *HMGCR* mRNA was measured by QPCR, normalized to GAPDH expression and expression was calculated as a fraction of that measured in cells transfected with NT siRNA (paired *t*-test, n = 3 independent transfections; *P < 0.05; **P < 0.005 compared to cells transfected with NT siRNA). Images presented in this panel were cropped to remove surrounding white space.

We first confirmed that HMGCR is highly expressed in ovarian cancer cell lines. HMGCR expression was higher in all 12 cell lines examined, than in normal ovarian epithelial cells (Fig. 1a,b). This included Ovcar-4, COV-318, COV-362, FuOv1 and Ovsaho cells which have recently been recognized as the most authentic cell line models of high-grade serous ovarian cancer (HGSOC)^30^. To determine if dysfunction of *TP53* might contribute to altered HMGCR expression in ovarian cancer cells, we ectopically expressed wild-type and R248W, R175H, and R273H gain-of-function p53 variants in SkOv-3 ovarian cancer cells which lack endogenous p53 protein^31^. P53 was not detected in the SkOv-3 cells transfected with the vector (Fig. 1c). Transfection with all three plasmids encoding *TP53* and its variants led to increased levels of HMGCR compared to cells transfected with the vector (Fig. 1c). To determine if pre-existing mutations in *TP53* also led to increased expression of *HMGCR*, we inhibited the expression of *TP53* in Ovcar-3 cells which contain a mutation in *TP53* encoding R248Q^32^. Four different siRNA directed to *TP53* mRNA significantly decreased both levels of p53 and HMGCR protein and, consistent with *TP53* regulating *HMGCR* expression, *HMGCR* mRNA was also reduced (Fig. 1d).

**Figure 2:**
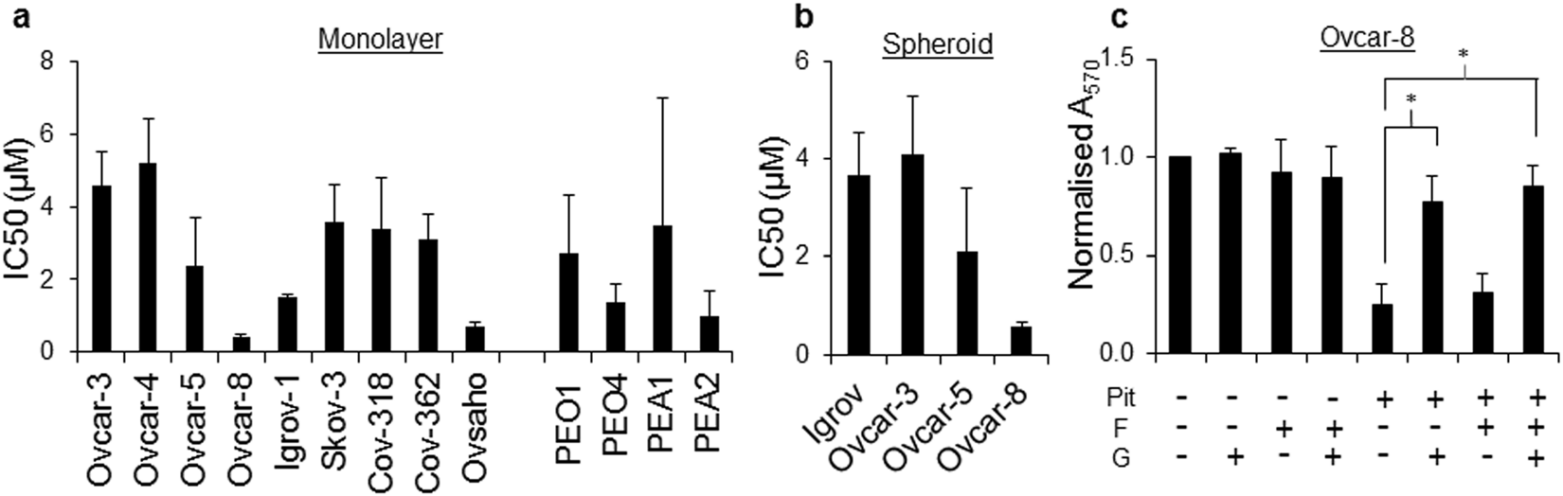
Pitavastatin inhibits the growth of cultures of ovarian cancer cells. The effect of pitavastatin was assessed in cell growth assays (mean IC_50_ ± S.D., n = 3–5 biological replicates) using monolayer (**a**) and spheroid cultures (**b**). (**c**) The growth inhibitory effects of pitavastatin (1 μM) on Ovcar-8 monolayer cultures were suppressed (*paired *t*-test, *P* < 0.01) by geranylgeraniol (G, 10 μM) but not farnesol (F, 10 μM).

**Figure 3:**
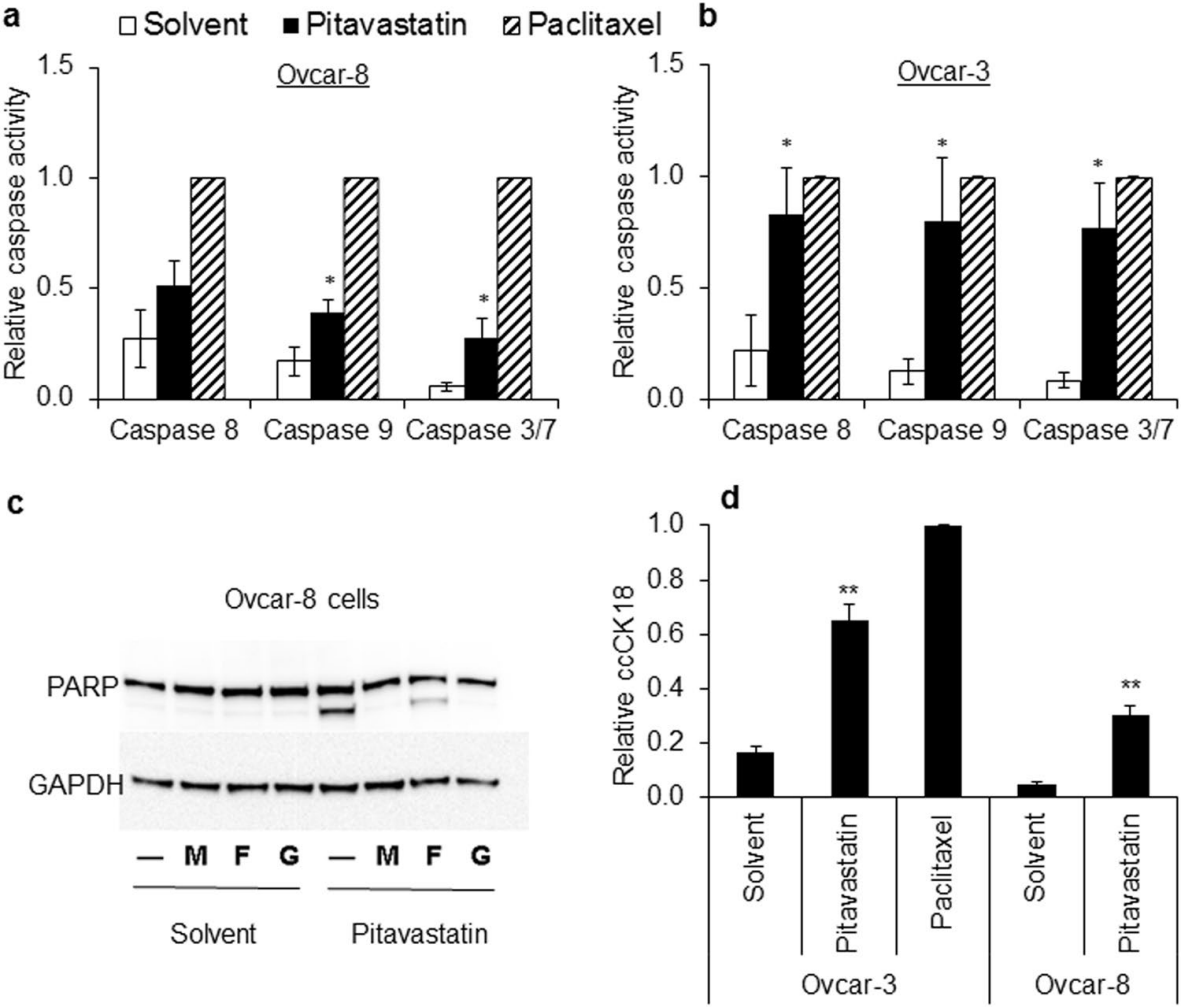
Pitavastatin induces apoptosis. (**a**,**b**) Pitavastatin (1 μM (5 × IC_50_, chosen to ensure a synchronous response), for 48 h) increased caspase 8, 9 and 3/7 activities in Ovcar-8 cells (**a**) and Ovcar-3 cells (**b**) compared to cells treated with an equal concentration of DMSO solvent used to dissolve pitavastatin (paired *t*-test, *P < 0.05, n = 3 biological replicates; results expressed as a fraction of that measured after exposure to paclitaxel). (**c**) Pitavastatin (1 μM, 48 h, n = 3 biological replicates) caused PARP cleavage in Ovcar-8 cells and this was suppressed by geranylgeraniol (G, 10 µM) and mevalonate (M, 100 µM) but not farnesol (F, 10 µM). Images presented in this panel were cropped to remove surrounding white space. (**d**) Pitavastatin increased the release of ccCK18 into cell culture supernatant of both Ovcar-3 cells and Ovcar-8 cells compared to cells treated with solvent (paired *t*-test, **P < 0.005, n = 3 biological replicates, normalized to ccCK18 release caused by paclitaxel exposure).

Pitavastatin inhibited the growth of a panel of ovarian cancer cells, including those considered most likely to represent HGSOC, grown as a monolayers (IC_50_ = 0.4–5 μM; Fig. 2a) or as spheroids (IC_50_ = 0.6–4 μM; Fig. 2b). This activity was reversed by the addition of the isoprenoid geranylgeraniol (Fig. 2c). Pitavastatin was able to inhibit the growth of cultures of cells which were relatively resistant to carboplatin, raising the possibility that pitavastatin might be useful in the treatment of drug-resistant disease (Fig. S1). Strikingly, when we compared cell lines derived from patients before (PEA1, PEO1) or after (PEA2, PEO4) the onset of clinical drug resistance^33^, pitavastatin was at least as potent in the chemotherapy-resistant cells as the chemotherapy-sensitive ones, suggesting pitavastatin may be useful to treat chemotherapy-resistant disease (Fig. 2a). Pitavastatin induced apoptosis, evidenced by the increased activity of executioner caspases-3,7 as well as caspase-8 and caspase-9 in two separate cell lines, and induced PARP cleavage (Fig. 3a–c). The increase in PARP cleavage was also prevented by inclusion of mevalonate or geranylgeraniol but not by farnesol, suggesting that pitavastatin, like simvastatin^20^, even when used at these relatively high concentrations causes cell death by inhibiting the HMGCR pathway. Furthermore, pitavastatin increased the secretion in two cell lines of caspase-cleaved cytokeratin 18 (ccCK18, Fig. 3d), a potential biomarker of tumour cell apoptosis in cancer patients^34^.

**Figure 4:**
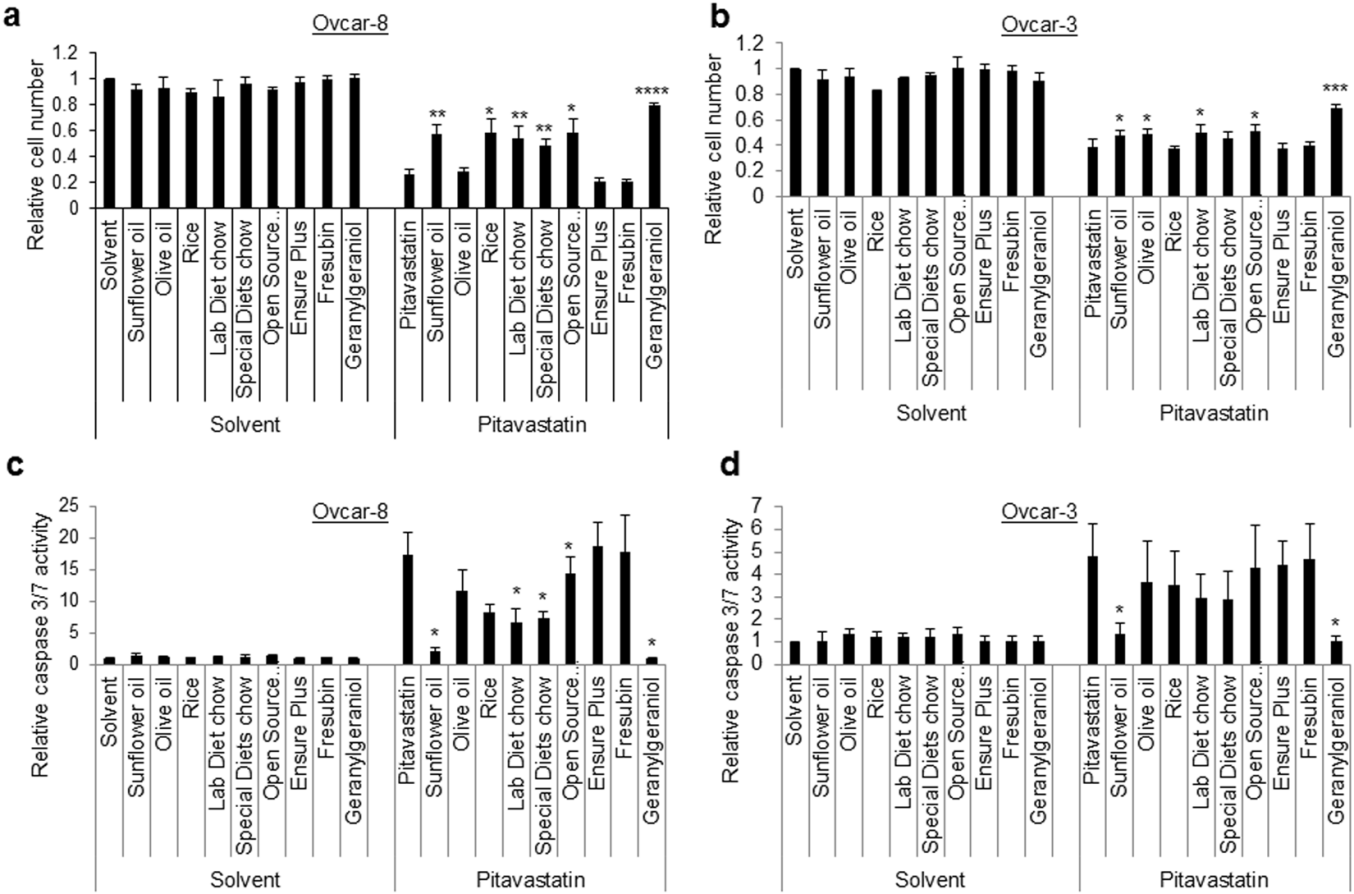
Ovcar-8 (**a**,**c**) or Ovcar-3 (**b**,**d**) cells were exposed to pitavastatin (1 μM) in the presence of solvent extracts (0.06% v/v) from the indicated human foodstuffs and mouse chow from different suppliers. (**a**,**b**) The number of cells remaining (mean ± S.D., expressed as a fraction of cells treated with solvent alone, n = 3–5 biological replicates) after 72 h was determined by staining with SRB. The number of cells was significantly different to cells exposed to pitavastatin alone where indicated (paired *t*-test, *P < 0.05; **P < 0.005; ***P < 1 × 10^−4^; ****P < 5 × 10^−6^). (**c**,**d**) Cells were exposed to pitavastatin and the solvent extracts as above and caspase 3/7 activity measured (mean ± S.D., n = 3 biological replicates, *significantly different from cells treated with pitavastatin alone, paired *t*-test, *P* < 0.05).

These studies prompted us to evaluate the activity of pitavastatin in a xenograft model. In a preliminary study (Fig. S2), tumours in animals treated with pitavastatin calcium twice daily were 85% of the volume of tumours in animals treated with vehicle, even though the tumours recovered from the animals at the end of the experiment contained 8–22 µM pitavastatin. We considered the possibility that dietary sources of geranylgeraniol might have bypassed the inhibition of HMGCR by pitavastatin. Mouse chow contains significant quantities of fat and several human food stuffs (e.g. rice, oils) have also been shown to contain geranylgeraniol^35–37^. To test whether this could interfere with the cytotoxicity of pitavastatin, we prepared organic solvent extracts of different mouse chows and human foods and found that many, particularly sunflower oil, suppressed the activity of pitavastatin in cell growth assays as well as in caspase 3/7 assays in two separate cell lines (Fig. 4). Extracts from mouse chow also inhibited the activity of pitavastatin. To demonstrate the presence of geranylgeraniol in the extracts, the sunflower extracts were analysed by GC-MS. We confirmed the previously reported^37^ presence of geranylgeraniol derivatives (Fig. S3). These data suggested that diet might have suppressed the activity of pitavastatin in our initial xenograft studies and may also have contributed to the lack of efficacy of statins in clinical trials.

**Figure 5:**
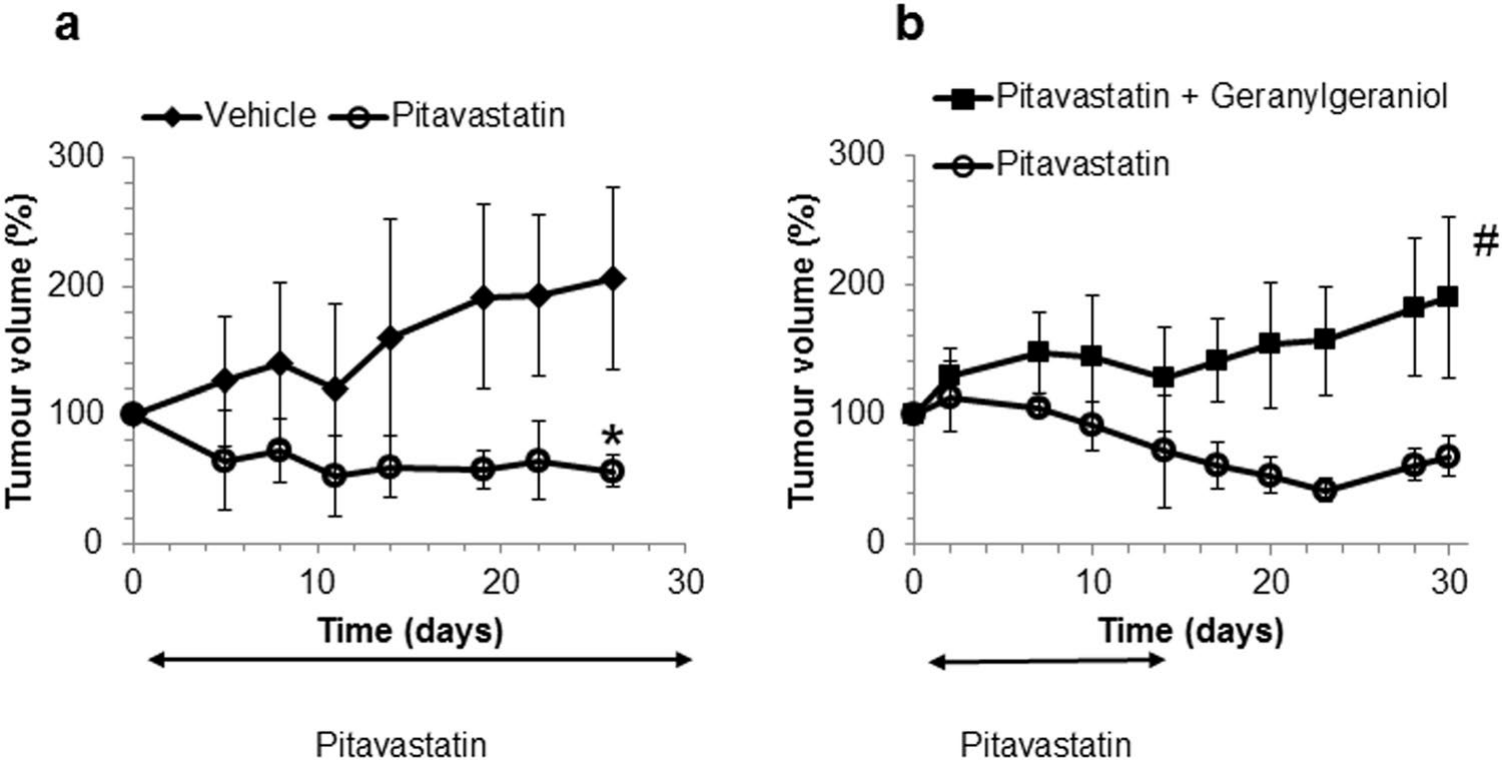
(**a**) Nude mice maintained on a diet of Ensure Plus and bearing subcutaneous Ovcar-4 tumours (mean volume 150 mm^3^, 6 animals per study arm) were treated twice daily with vehicle or pitavastatin-calcium (59 mg/kg, p.o.). After 28 days, tumour volume was significantly different in the mice receiving pitavastatin (*P < 0.005, *t*-test with Welch’s correction). Animal body weight increased throughout the course of the dosing period and no significant adverse events were observed. (**b**) Nude mice bearing subcutaneous Ovcar-4 tumours (mean volume 220 mm^3^, 4 animals per study arm) were treated twice daily with pitavastatin-calcium (59 mg/kg, p.o.) for days 1–14 while being provided a diet of either Ensure Plus or Ensure Plus supplemented with 0.14 mg/mL geranylgeraniol. Animal body weight increased throughout the course of the dosing period and no significant adverse events were observed. Mice were returned to regular chow on day 16. After 28 days, tumour volume was significantly larger in the animals receiving dietary geranylgeraniol (^#^ *P* < 0.05, *t*-test with Welch’s correction).

We looked for an alternative food free of isoprenoids that could be used in these settings. Organic solvent extracts of the complete oral liquid food supplements Ensure Plus and Fresubin did not supress the activity of pitavastatin in cell growth or caspase assays (Fig. 4). In a xenograft study in which mice bearing Ovcar-4 tumours were fed Ensure Plus in place of regular chow, pitavastatin (59 mg/kg, b.i.d., p.o.) caused significant tumour regression (56 ± 12% of initial tumour volume, n = 7; *P* < 0.005; Fig. 5a) whereas tumours in mice which received the vehicle continued to grow (205 ± 71% of initial volume). Ki-67 staining was observed in 59 ± 12% of the tumour cells recovered from animals which received vehicle, but significantly fewer cells (34 ± 16%, *P* < 0.05) were Ki-67 positive in the residual tumour tissue recovered from mice which received pitavastatin. To evaluate whether dietary geranylgeraniol could suppress the activity of pitavastatin, nude mice bearing Ovcar-4 tumours were maintained on Ensure Plus or Ensure Plus supplemented with 0.14 mg/mL geranylgeraniol. Both sets of mice where subsequently treated with pitavastatin. In the absence of dietary geranylgeraniol, pitavastatin caused tumour regression (61 ± 13% of initial volume, n = 4) whereas tumours in mice who received dietary geranylgeraniol and pitavastatin continued to grow and were significantly larger (182 ± 52% of initial volume, n = 4, *P* < 0.05; Fig. 5b) than the mice maintained on unsupplemented Ensure Plus. Tumours were collected 8 hours after the last drug administration to measure the trough pitavastatin concentration. The concentration of pitavastatin in tumours excised from animals receiving pitavastatin and geranylgeraniol was 3.1 μM, significantly lower than that measured in the Ovcar-3 tumours (Supplementary Figure 2). The drug was undetectable in the small residual Ovcar-4 tumours that had regressed in the animals treated with pitavastatin alone.

## Discussion

Our previously published study^20^ suggests that the physicochemical and pharmacological profile of pitavastatin makes it the statin most likely to be efficacious in oncology clinical trials. We have shown that pitavastatin induces apoptosis in ovarian cancer cells. Statins have demonstrated activity in several other cancer types^13^, suggesting that pitavastatin may useful to treat a range of cancers. Indeed pitavastatin has been shown to inhibit the growth of glioblastoma cells in xenograft studies in mice^38^. However, we report for the first time that restriction of diet to eliminate geranylgeraniol allows statins to induce tumour regression.

Pitavastatin was effective in all of the cell lines tested, with potencies differing by approximately 10-fold between different cell lines. This may reflect a fundamental role of the mevalonate pathway in ovarian cancer cell biology. In support of this, we observed that increased expression of both wild-type and gain-of-function variants of *TP53* led to increased HMGCR expression. Together with the wide spread dysregulation of *TP53* in ovarian cancer^7^, and the detection of HMGCR in a large proportion of ovarian cancer tumours^8^, this suggests that a significant proportion of ovarian cancer patients may be candidates for treatment with pitavastatin. The selection of patients for statin therapy may be further guided by gene expression profiles which predict statin sensitivity^39^. Of particular interest, we found that pitavastatin retained its activity in matched cells derived from patients before and after the onset of clinical drug resistance. This raises the possibility that statins may be useful to treat patients with chemotherapy-resistant disease, a patient population currently lacking adequate therapy.

We found that supplementing the diet of animals with geranylgeraniol prevented the tumour regression caused by pitavastatin. Geranylgeraniol is likely to be converted into its pyrophosphate inside cells because this is the substrate for geranylgeranyl transferases. We administered the geranylgeraniol rather than its pyrophosphate form because we anticipated that it is unlikely that the pyrophosphate would be orally bioavailable, due to reduced membrane permeability compared to the free alcohol and hydrolysis of the phosphoester in the acidic environment of the stomach. Although methods to measure geranylgeranyl pyrophosphate have been developed^40^ additional methods to evaluate the bioavailability of different geranylgeraniol derivatives are needed.

Although we have used pitavastatin at a relatively high dose/concentration, there is good reason to believe its activity is mediated by inhibition of the mevalonate pathway. Firstly, products of the mevalonate pathway, geranylgeraniol and mevalonate, but not farnesol, reverse the activity of pitavastatin and other statins^20^ *in vitro*; we further show here that geranylgeraniol inhibits the activity of pitavastatin in animal studies. Farnesol presumably fails to do the same because it requires the production of 5 carbon isoprenoids for it to be converted into geranylgeraniol, and this is blocked by pitavastatin. Secondly, several studies, including our own unpublished observations, show that statins (lovastatin, simvastatin, fluvastatin, pitavastatin) interact synergistically with inhibitors of geranylgeranyl diphosphate synthase^41^ or farnesyldiphosphate synthase^42–45^. For two drugs to act synergistically their molecular targets must lie on interacting pathways, so synergy between statins and other inhibitors of the mevalonate pathway is consistent with pitavastatin acting through inhibition of the mevalonate pathway. Thus, the activities of statins are enhanced by drugs which inhibit the mevalonate pathway and suppressed by addition of metabolites produced by the mevalonate pathway, strongly supporting the contention that pitavastatin causes cell death through inhibition of the mevalonate pathway.

The effect of diet on the activity of pitavastatin which we have observed may explain the discrepancies between positive preclinical studies of statins in cancer, retrospective clinical studies in which statins have been associated with modest improvements in cancer outcome, and prospective clinical trials which have not yet demonstrated widespread efficacy. In retrospective studies of patients receiving statins for hypercholesterolaemia, patients may have received dietary advice to reduce fat intake but it is unlikely that all sources of dietary geranylgeraniol were eliminated. Analysis is also complicated because patients in these studies received different statins at different doses. The relatively large cohorts of patients in these retrospective trials, together with reduced dietary isoprenoids may have allowed modest survival benefits to be detected. We have shown continual inhibition of HMGCR is necessary to cause cancer cell death^20^. The failure to ensure continual inhibition of HMGCR, particularly a problem with a low dose of a statin with a short half-life and which is administered once daily, may have also prevented more significant clinical results from being obtained. We consider that successful prospective clinical trials of statins in cancer can best be designed by evaluating relatively high doses of a potent lipophilic statin with an adequate half-life, which is administered at a suitable dosing frequency to ensure continual inhibition of HMGCR, and dietary sources of geranylgeraniol or other mevalonate pathway metabolites should be controlled. No trials published to date have addressed all three factors, potentially explaining the lack of notable clinical success with statins in oncology to date. We have identified one potential diet that could be used in clinical trials for the duration of statin treatment – in our studies we saw tumour regression within 2 weeks. It is feasible that patients could be restricted to a diet of Ensure Plus for such a period, although it is clearly desirable to identify additional foods that do not interfere with the activity of statins. We consider that pitavastatin, which has gained regulatory approval in both the US and the European Union for the treatment of hypercholesterolaemia, is the most appropriate statin for clinical oncology studies because of its lipophilicity, relatively long half-life and oral bioavailability^29^. Statins have shown efficacy *in vitro* in a broad range of cancer types^13^, suggesting that pitavastatin warrants preclinical and clinical studies in these cancers but taking into account the factors which we have identified. This may lead to the use of statins in a range of cancer types.

We administered pitavastatin at a relatively high dose (½ MTD). This is significantly higher than the dose of pitavastatin (up to 4 mg) currently used to treat hypercholesterolaemia but this dose allowed us to achieve microMolar concentrations of drug in the tumours. An issue that may limit the use of statins, particularly at high doses, is that statins have been associated with myopathy^29^ and in some cases this can result in rhabodomyolysis. It will be important to evaluate in clinical trials whether a suitable dose can be found which is efficacious without unacceptable toxicity. However, clinical trials of high dose statins have not recorded myopathy within 1–2 weeks of commencing therapy, although only small numbers of patients have been assessed. Cycles of brief (1–2 weeks) high-dose pitavastatin therapy may minimize the incidence if myopathy. Clinical studies, designed to the criteria which we have identified, are needed to evaluate escalating doses of pitavastatin to define the therapeutic window for pitavastatin in an oncology setting.

Lastly, although the goal of these studies was to explore the potential for pitavastatin to be used to treat cancer, our observations have broader implications. There is currently particular interest in targeting several metabolic pathways to treat cancer and clinical trials of such drugs are underway^46^. We suggest that clinical trials of drugs targeting metabolic pathways should be designed to take into account the potential for patients to ingest metabolites on the pathway which is being targeted and which could potentially impair drug efficacy. It seems prudent to assess this in preclinical studies, similar to those we report here, evaluating different dietary sources of these metabolites prior to conducting clinical trials.

## Materials and Methods

### Cell Culture

Cell lines were obtained from either ATCC, ECACC, NIH or JCRB. Human ovarian cancer cell lines (A2780, cisA2780, Igrov-1, Skov-3, Ovcar-3, Ovcar-4, Ovcar-5,Ovcar-8, COV-318, COV-326 Ovsaho, PEA1, PEA2, PEO1, PEO4), human ovarian epithelial (HOE) cells and primary human foreskin fibroblasts (HFF) were grown in RPMI supplemented with 10% FCS, 50 U/mL Penicillin/Streptomycin and 2 mM Glutamine. The NL20 cell line was grown in Ham’s F12 medium supplemented with 4% FCS, 1.5 g/L sodium bicarbonate, 2.7 g/L glucose, 2 mM glutamine, 0.1 mM non-essential amino acids, 0.005 mg/mL insulin, 10 ng/mL epidermal growth factor, 0.001 mg/mL transferrin and 500 ng/mL hydrocortisone. Cells were incubated at 37 °C and 5% CO_2_ and tested for mycoplasma.

### Cell Growth Assays

Cell growth assays were performed as previously described^47^. Cells (5000 cells/well except for A2780, cisA2780 and Ovcar-8, where 2000 cells/well were used) were seeded in 96-well plates in 80 μL of growth medium. After 24 h, 20 μL of 18 different concentrations of drug or food extract at 5 times the required concentration or a drug solvent control was added to the cells. After incubation for a further 72 h, the growth medium was removed and the cells in each well were fixed in 100 μL 10% trichloroacetic acid (TCA) on ice for 30 min. The plates were washed three times in water, dried and stained in 0.4% sulforhodamine B (SRB) in 1% acetic acid for 30 minutes. After washing three times in 1% acetic acid and drying, the dye was solubilised in 100 μL 10 mM Tris (pH 10) and the absorbance at 570 nm (A_570_) was determined using a BioTek Synergy 2 multi-mode microplate reader. For experiments evaluating the ability of isoprenoids to reverse the effect of pitavastatin, cells were simultaneously exposed to either 100 μM mevalonate, 10 μM farnesol, 10 μM geranylgeraniol or DMSO.

### Three-Dimensional Spheroid Culture and ATP Assays

Ovcar-3 (5 × 10^5^ cells/mL), Ovcar-5 (1 × 10^6^ cells/mL), Ovcar-8 (1.25 × 10^5^ cells/mL) and Igrov-1 (2 × 10^6^ cells/mL) cells were prepared as cell suspensions in growth medium. One 20 μL drop of cell suspension was added to each inner ring of the lids of 48-well plates. Outer rings contained a 20 μL drop of growth medium and 300 μL of sterile water was added to each well beneath the rings to maintain a humid atmosphere and minimise evaporation. The lids were inverted over the plates and incubated for 7 days. After 1 week, the spheroids were exposed to 5 μL growth medium containing pitavastatin or solvent and incubated for a further 72 h. The spheroids were then collected using a wide bore pipette into opaque-walled multiwell plates containing 20 μL of PBS and an equal volume of CellTiter-Glo reagent was added in order to measure ATP. Spheroid lysis was confirmed by microscopy to ensure penetration of the reagent into the spheroid. Luminescence was measured using a BioTek Synergy 2 multi-mode microplate reader.

### ImmunoBlotting

For western blotting, 5 × 10^5^ cells were exposed to pitavastatin in the presence or absence of 100 μM mevalonate, 10 μM farnesol, 10 μM geranylgeraniol or DMSO (these concentrations were found in preliminary experiments to be both soluble in aqueous media and not cytotoxic). After 48 h, the cells were collected, washed in PBS and lysed in a modified RIPA containing protease inhibitors^48^. Protein concentration was determined using a BCA assay and samples normalized to load equal mass of protein in each lane (10–20 μg). After electrophoresis and transfer to PVDF, membranes were incubated with anti-PARP antibody (1/1000, #9542, Cell Signaling), anti-HMGCR [EPR1685(N)] antibody (1/1000, AB174830, Abcam), p53 (1/5000, Ab179477, Abcam) or anti-GAPDH antibody (1/5000, MAB374, Millipore) as a loading control. Bands were quantified using AlphaView SA (ProteinSimple) and normalized to GAPDH.

### Caspase 3/7, 8 and 9 Assays

Ovcar-8 (2000 cells/well) and Ovcar-3 (5000 cells/well) cells were incubated in 80 μL growth medium in a 96-well plate for 24 h. Cells were then supplemented with 20 μL growth medium containing pitavastatin, paclitaxel or solvent. In other experiments, Ovcar-8 and Ovcar-3 cells were exposed to pitavastatin for 48 h, then 25 μL of Caspase-Glo 3/7, 8 or 9 reagent (Promega) was added and cells were incubated for 30 minutes at room temperature in the dark. A BioTek Synergy 2 multi-mode microplate reader was used to measure luminescence.

### M30 CytoDeath ELISA

Ovcar-8 (2000 cells/well) or Ovcar-3, (5000 cells/well) were incubated in 100 μL growth medium in a 96-well plate for 24 h and then exposed to pitavastatin at five times the IC_50_ measured in cell growth assays, paclitaxel (50 nM) or solvent. After 72 h, the supernatant was collected and centrifuged at 10,000 g for 10 min at 4 °C. The M30 CytoDeath ELISA (Peviva) was completed following the manufacturer’s instructions. A_450_ was measured using a BioTek Synergy 2 multi-mode microplate reader.

### siRNA Studies

Si-genome p53 siRNA (p53#1, GAAAUUUGCGUGUGGAGUA; p53#2, GUGCAGCUGUGGGUUGAUU; p53#3, GCAGUCAGAUCCUAGCGUC; p53#4, GGAGAAUAUUUCACCCUUC), a non-targeting siRNA NT-1 or SMARTpool (Dharmacon) comprising the same oligos were used to inhibit the expression of *TP53*. Ovcar-3 cells ((5000 cells/well) were plated in a 96 well plate in 80 µl of antibiotic-free growth medium per well). The following day the cells were transfected with 0.1% Dharmafect-1 and 100 nM siRNA as previously described^49^. The next day, the media were replaced with 100 µL of fresh antibiotic-free growth media. Knockdown of *TP53* expression was assessed by immunoblotting and QPCR.

### Transient expression of p53

Expression studies were approved by the Keele genetic modifications of microorganisms committee. SkOv-3 cells (32,000 cells per well of a 24 well plate) were transfected with 0.1 μg CMV-Neo-Bam (vector), pCMV-Neo-Bam p53 wt, pCMV-Neo-Bam p53 R175H, pCMV-Neo-Bam p53 R273H, pCMV-Neo-Bam p53 R248W (Addgene) and 0.2 μL of Lipofectamine 2000 as previously described^47^. Protein expression was measured by immunoblotting.

### Xenograft studies

Ovcar-3 xenograft studies were completed by Charles River Discovery Research Services in Morrisville, North Carolina. Animal studies at Charles River were approved by Charles River Institutional Animal Care and Use Committee and the studies complied with all relevant guidelines and regulations. Female CB17 severe combined immunodeficiency (SCID) mice at an age of 8–12 weeks were subcutaneously injected in the flank with 1 mm^3^ Ovcar-3 tumour fragments. When tumours reached an average of 100 mm^3^, animals were assigned randomly either to the drug or vehicle arm of the trial. A separate cohort received paclitaxel as a positive control. Mice received either 79 mg/kg (representing ½ maximum tolerated dose) pitavastatin calcium suspension p.o. prepared in 0.5% carboxymethyl cellulose in sterile water or vehicle every 12 h for 33 days (10 mice per group).

A subsequent study with Ovcar-4 was approved by Keele Animal Welfare and Ethical Review Body and conducted under a license granted under the Animal (Scientific Procedures) Act 1986 following institutional guidelines. For Ovcar-4 xenograft studies, 4 week old female NCR Nu/Nu female mice (Envigo) were injected s.c. with 3 million Ovcar-4 cells suspended in 50% cultrex in RPMI. When the tumours were established, animals were adapted for one week to a diet of Ensure Plus *ad libitum* and regular chow was withdrawn. Pairs of animals with comparable size tumours were assigned randomly to either arm of the study and received either 59 mg/kg (representing ½ maxium tolerated dose) p.o. pitavastatin calcium suspension (8 mg/mL) twice daily in 0.5% carboxymethyl cellulose in sterile water or the drug vehicle. Each arm of the study comprised 6 animals. Tumour volume and animal weight was monitored twice weekly. The investigators were not blinded to the drug treatment.

In experiments to measure the effect of geranylgeraniol, animals with established tumours were again adapted to a diet of Ensure Plus one week before the start of the experiment. The animals were randomized to either arm of the study as described above (4 animals per arm). All animals received pitavastatin as described above from days 1–14. The animals received either Ensure Plus or Ensure Plus freshly supplemented daily with 0.14 mg/mL geranylgeraniol (Santa Cruz) from day 1-day 16. This concentration of geranylgeraniol was estimated to be equivalent (per kg bodyweight) to that which has previously been delivered to rats^50^. On day 17, all animals were switched back to regular mouse chow.

### Extraction and Detection of Pitavastatin in Ovcar-3 Tumours

50–200 mg of each tumour sample was homogenised in 200 μL PBS using a Dounce homogeniser. The resulting suspension was centrifuged at 10,000 g for 10 min at 4 °C and the supernatant was collected. Supelclean LC-18 solid phase extraction (SPE) columns (SupelCo) were conditioned with 3 mL 99% methanol, followed by 3 mL deionised water, and then 3 mL 0.5 M monobasic potassium phosphate. The sample was then applied and the column washed with 3 mL 0.5 M monobasic potassium phosphate and 3 mL deionised water. Pitavastatin was eluted with 3 mL methanol and the eluate dried. The residue was re-suspended in 100 μL 99% methanol for HPLC analysis or growth medium for cell growth assays.

For HPLC analysis, samples were diluted in acetonitrile and a 20 μL sample analysed on a reverse-phase C18 column (Metlab Supplies) with a mobile phase of acetonitrile:water with 0.1% formic acid (65:35) and 1 mL/min flow rate. Pitavastatin was detected with an excitation of 245 nm and an emission of 420 nm and quantified using standards treated in the same way.

Alternatively, a bioassay was performed in which a range of concentrations of the extracts were added to Ovcar-8 cells and the effect of the extract in cell growth assays, performed as described above, was assessed, In parallel, the effect of known concentrations of pitavastatin was measured and used to estimate the concentration of pitavastatin in the extracts.

### Extraction and Detection of Pitavastatin in Ovcar-4 Tumours

Tissue from tumour drug accumulation studies were prepared for analysis by homogenisation in a Dounce homogenizer (4 μL per mg of tissue) in deionized water. Three parts isopropanol containing 1 µg/mL internal standard simvastatin was added to one part sample homogenate and vials were vortexed for 30 seconds and placed on ice. Samples were centrifuged (8000 g, 20 minutes, 4 °C) and supernatant collected. The extraction efficiency of pitavastatin and internal standard simvastatin was 72% and 97% respectively. The HPLC-MS/MS system used for sample analysis consisted of an autosampler model Dionex Ultimate 3000, and an Applied Biosystems 3200 QTrap mass spectrometer (Thermo, UK) utilising a Turbo Ionspray source. The precursor mass-to-charge ratio, collision-induced mass shift and optimal detection settings for pitavastatin and internal standard simvastatin were obtained from previous publications^51, 52^. Chromatographic separation was performed at 25 °C on a Hypersil Gold reverse-phase C-18 3 µM column (50 × 2.1 mm i.d., ThermoFisher, UK). An isocratic mobile phase was utilised during each 10 minute run at a flow rate of 0.3 mL/minute and consisted of 70% methanol, 30% MS grade water and 0.05% formic acid. The retention time of pitavastatin and internal standard simvastatin was 3.31 minutes and 4.92 minutes, respectively, and the lowest limit of pitavastatin quantification was 30.6 ng/mL. The high concentration quality controls (HQC, 1900 ng/mL, n = 3) had a mean intra-day accuracy and precision of 97.5% and 95.5%, respectively. The medium concentration quality controls (MQC, 600 ng/mL, n = 3) had a mean intra-day accuracy and precision of 94.7% and 97.4%, respectively. The low concentration quality controls (LQC, 60 ng/mL, n = 3) had a mean intra-day accuracy and precision of 88.3% and 95.0%, respectively. All QCs tested gave higher than 85% accuracy and precision and the linear range for pitavastatin quantification was 30.6–2000 ng/mL.

### Preparation of food extracts and analysis by GCMS

Kinuhikari rice^36^ was obtained from the cultivator and oils were purchased from food stores in the UK. Ensure Plus Raspberry Flavour Drink and Fresubin 2 kcal Vanilla Flavour Drink were donated by a local pharmacy. Mouse chows included Lab Diet NIH 31 0045117 from Charles River in North Carolina, Special Diets Services 801960 BK001(E) from Keele University in the UK and Open Source Diets D11112201 from University of British Columbia in Canada. Solid foods (50 g) were homogenized in a food processor in 30 mL methanol and extracted by further additions of 30 mL chloroform and 30 mL methanol. The extracts were filtered and evaporated to dryness. Alternatively, liquid foodstuffs were transferred to a separating funnel and extracted with 60 mL methanol and 30 mL chloroform. The lower lipid phase was evaporated to dryness. The residues were dissolved in 25 mL ethanol and hydrolysed with 25 mL 5 M potassium hydroxide at 56 °C for 1 h. After cooling, and neutralization with 25 mL 5 M hydrochloric acid the solution was partitioned with 120 mL n-hexane, 30 mL water and 30 mL ethanol. The upper organic phase was evaporated to dryness and dissolved in DMSO for analysis.

### Chemical Analysis: Gas Chromatography – Mass Spectrometry

Dried sunflower oil extract were re-dissolved in hexane before analysis. Chemical analysis of these sunflower extracts (2 μL) was carried out on an Agilent Technologies 7890 N Network GC with a split/splitless injector at 250 °C in split mode (ratio 100:1), a Agilent VF-5HT capillary column (30 m × 0.25 mm ID, 0.1 μm film thickness) and coupled to an Agilent 5975 Network Mass Selective Detector. The GC was coupled to a computer and data processed with Agilent Chemstation software. Elution was carried out with helium at 1 mL/min. The oven temperature was programmed to be held at 35 °C for 2 minutes then ramped to 300 °C at 5 °C/min and then from 300 °C to 350 °C at 10 °C/min. The mass spectrometer was operated in Electron Ionisation mode at 70 eV, scanning from 40–800 amu at 1.5 scans s^−1^. Compounds were identified using a library search (NIST08) and the diagnostic fragmented ions.

### Statistical Methods

To analyse cell growth assays, Graphpad Prism was used to fit a 4 parameter Hill equation to the data. The statistical methods used for each experiment are presented in the relevant figure legends.

### Data availability

The datasets generated during and/or analysed during the current study are either included in the published article or are available from the corresponding author on reasonable request.

## Electronic supplementary material

Supplementary information
