# Supplementary material for: Dietary geranylgeraniol can limit the activity of pitavastatin as a potential treatment for drug-resistant ovarian cancer
Source: Sci Rep. 2017 Jul 14;7:5410. doi: 10.1038/s41598-017-05595-4 (PMC5511264; doi:10.1038/s41598-017-05595-4)
Supplement: Supplementary file 1 — Supplementary information [file 41598_2017_5595_MOESM1_ESM.pdf]

Supplementary Figures

Dietary geranylgeraniol can limit the activity of pitavastatin as a potential treatment for drug-resistant ovarian cancer.

Elizabeth de Wolf, Marwan Ibrahim Abdullah, Stefanie M. Jones, Karen Menezes, Darren M. Moss, Falko P. Drijfhout, Sarah R. Hart, Clare Hoskins, Euan A. Stronach, Alan Richardson.

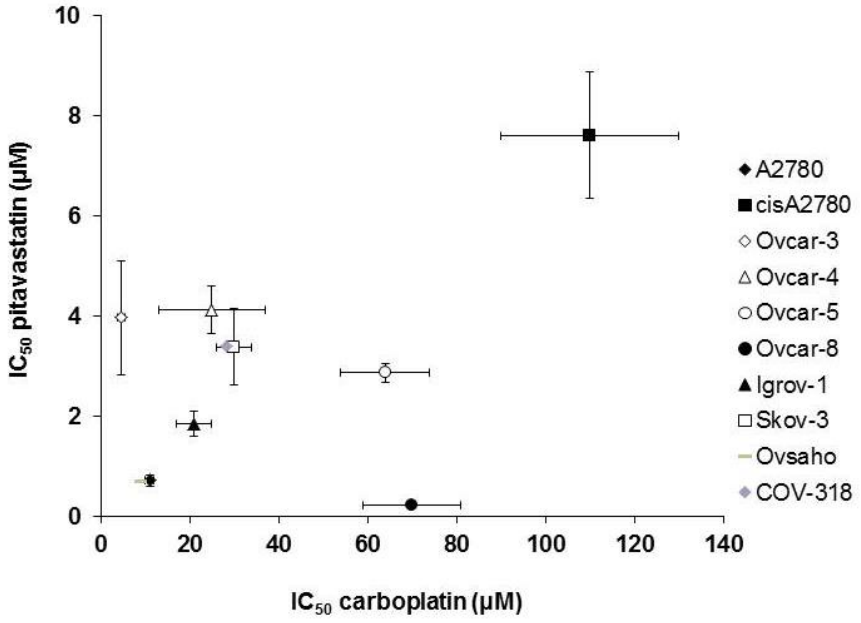

Supplementary Figure 1 Comparison of the sensitivity of ovarian cancer cells to carboplatin and pitavastatin. The activity of each compound was measured in cell growth assays and the IC_{50} determined. Linear regression analysis (r^2=0.25) indicated that the gradient did not differ significantly from zero.

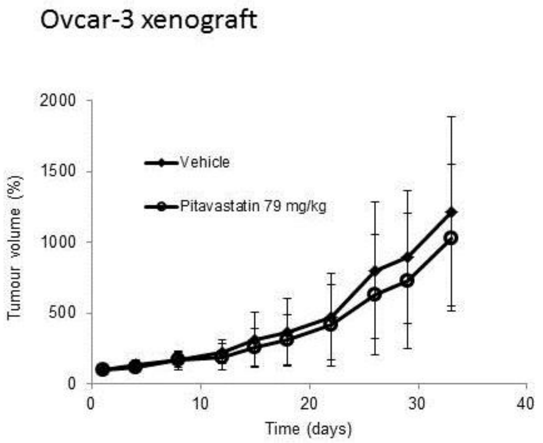

Ovcar-3 tumour xenograft	Pitavastatin concentration in tumour extract (HPLC) (µM)	Pitavastatin concentration in tumour extract (bioassay) (µM)
Group 1 #1-3	0	0
Group 2 #1	106	17
Group 2 #2	32	8
Group 2 #3	114	22

Supplementary Fig. 2 Preliminary xenograft experiment. SCID mice maintained on a diet of mouse chow bearing subcutaneous Ovcar-3 tumours (mean volume 100 mm^3, 10 animals per study arm) were treated twice daily with vehicle or pitavastatin-calcium (79 mg/kg, p.o.). After 33 days, tumour volume was not significantly different in the mice receiving pitavastatin. Tumours were excised and the drug concentration measured in 3 tumours was 84 ± 45 µM (measured by HPLC) and 14 ± 7 µM measured by a bioassay. The lower concentration in the latter assay may reflects the lower precision or there may be geranylgeraniol contaminating the extracts rendering the pitavastatin apparently less potent. Irrespective of this, both methods suggest microMolar concentrations of drug, well above the IC50, were measured in the tumours.

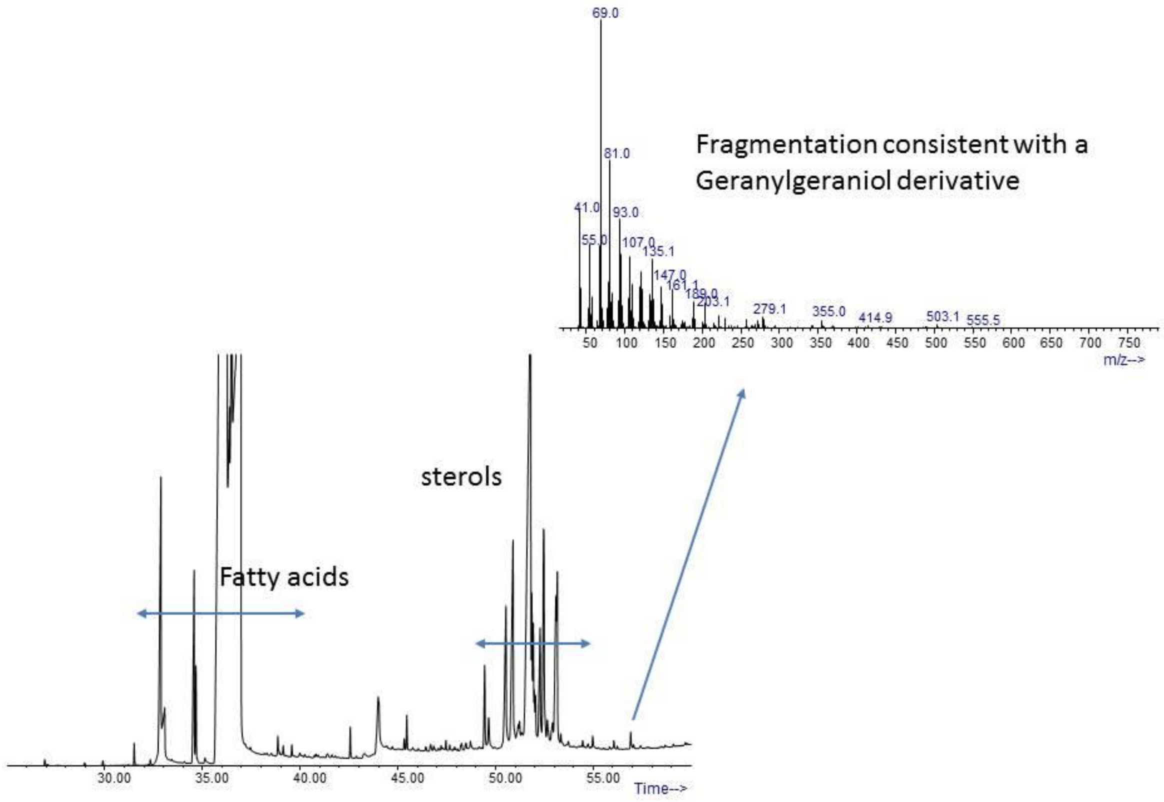

Supplementary figure 3. **Identification of geranylgeraniol derivatives in sunflower oil.** Solvent extracts of sunflower oil were analysed by GCMS. MS analysis indicates the presence of geranylgeraniol, but its relatively late elution after fatty acids and sterols indicates that it is a derivative of geranylgeraniol.
